# Skeletal manifestations in lymphatic diseases: nonenhanced magnetic resonance lymphography

**DOI:** 10.1186/s13244-026-02208-5

**Published:** 2026-03-27

**Authors:** Clément Cholet, Lise Minssen, Anne Miquel, Caroline Parlier-Cuau, Hedi Chekir, Lionel Arrivé

**Affiliations:** 1 Service d’Imagerie Médicale, Hôpital Saint-Antoine, Assistance Publique–Hôpitaux de Paris (AP-HP), Paris, France; 2 Paris Sorbonne Université, Faculté de Médecine, Paris, France; 3 Institut Curie, PSL Research University, Service de radiologie, F-92210, Saint-Cloud, France

**Keywords:** Generalized lymphatic anomalies, Gorham–Stout disease, Bone lesions, MRI, Magnetic resonance lymphography

## Abstract

**Abstract:**

The diffuse lymphatic diseases—generalized lymphatic anomalies (GLA) and primary lymphangiectasia—can present with both visceral and skeletal involvement. These diseases are life-threatening and can lead to malnutrition and immunodeficiency as consequences of impaired lymphatic function. Skeletal involvement has specific complications, including deformities, pain, fractures, septic arthritis, and osteomyelitis. The presence of bone involvement with bone lysis and cortical destruction is a marker of disease extension and severity. Identification of skeletal involvement may orient patients toward targeted therapies. On standard CT and MRI, bone lesions of lymphatic origin usually show no specific features, and etiological diagnosis can be difficult. Nonenhanced magnetic resonance lymphography (NEMRL) is a noninvasive and non-ionizing technique based on T2-weighted sequences that enables the visualization of the lymphatic circulation. In the setting of undetermined bone lesions, NEMRL may suggest a lymphatic origin by identifying extraosseous lymphatic anomalies or a communication between the bone lesion and the lymphatic system. Furthermore, NEMRL may determine the cause of the lymphatic anomalies and guide therapeutic options. The technique of NEMRL, the different imaging patterns of bone lesions of lymphatic origin, and the associated lymphatic anomalies identified by NEMRL that can orient the diagnosis and help in classifying lymphatic diseases are discussed.

**Critical relevance statement:**

Nonenhanced magnetic resonance lymphography is a non-ionizing, noninvasive tool to detect and characterize skeletal involvement in diffuse lymphatic diseases.

**Key Points:**

- Skeletal involvement in lymphatic disorders ranges from simple lymphangioma to bone destruction.
- NEMRL is noninvasive and allows the study of lymphatic system disorders.
- NERML evaluates lymphatic disease extension when assessing a lymphatic bone lesion.
- NEMRL can confirm the lymphatic origin of an undetermined bone lesion.
- NEMRL sometimes identifies communications between lymphatic bone lesions and extraosseous lymphatic anomalies.

**Graphical Abstract:**

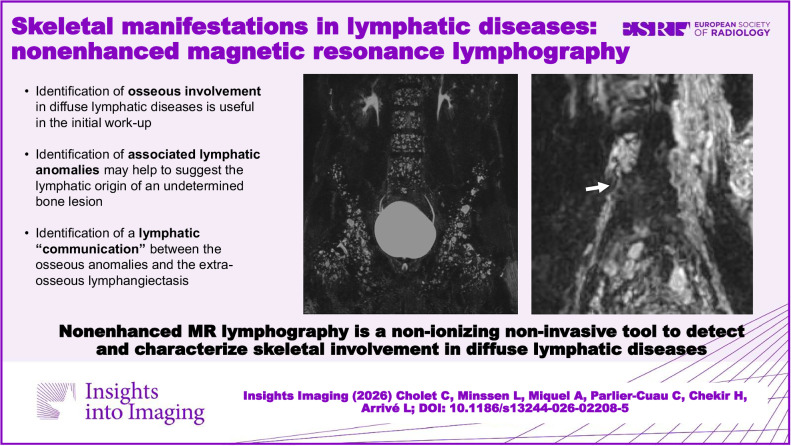

## Introduction

Lymphatic disorders, including generalized lymphatic anomalies (GLA) and primary lymphangiectasia, are rare diseases that may affect the entire lymphatic system. The most common findings are lymphangiectasis (lymphatic vessel dilation) and lymphangioma (a cyst-like lymphatic pocket) [1]. Although the majority of cases present exclusively with soft tissue and visceral involvement, some patients may also exhibit skeletal involvement. Lymphatic disorders must be recognized and treated, as they may be associated with life-threatening complications such as immunodeficiency, malnutrition, and hydroelectrolytic disorders. Skeletal involvement should be identified, as it may lead to specific complications, including bone and joint infections, fractures, and deformities [2]. The presence of bone involvement with bone lysis and cortical destruction is a marker for disease extension and severity. These anomalies have a broad range of phenotypic expression and severity, from benign lymphangioma to aggressive bone destruction.

Nonenhanced magnetic resonance lymphography (NEMRL) is a modern noninvasive and non-ionizing technique based on T2-weighted sequences. It allows the mapping of lymphatic anomalies throughout the body, and is of interest in the work-up of diffuse lymphatic disorders [3–6]. NEMRL, associated with conventional computed tomography (CT) and magnetic resonance imaging (MRI), may not only detect but also characterize intraosseous lesions of lymphatic origin. In this article, we present the spectrum of NEMRL imaging features that can be identified in lymphatic diseases with skeletal involvement.

## Bone lymphatic system anatomy and physiopathology

**Fig. 1:**
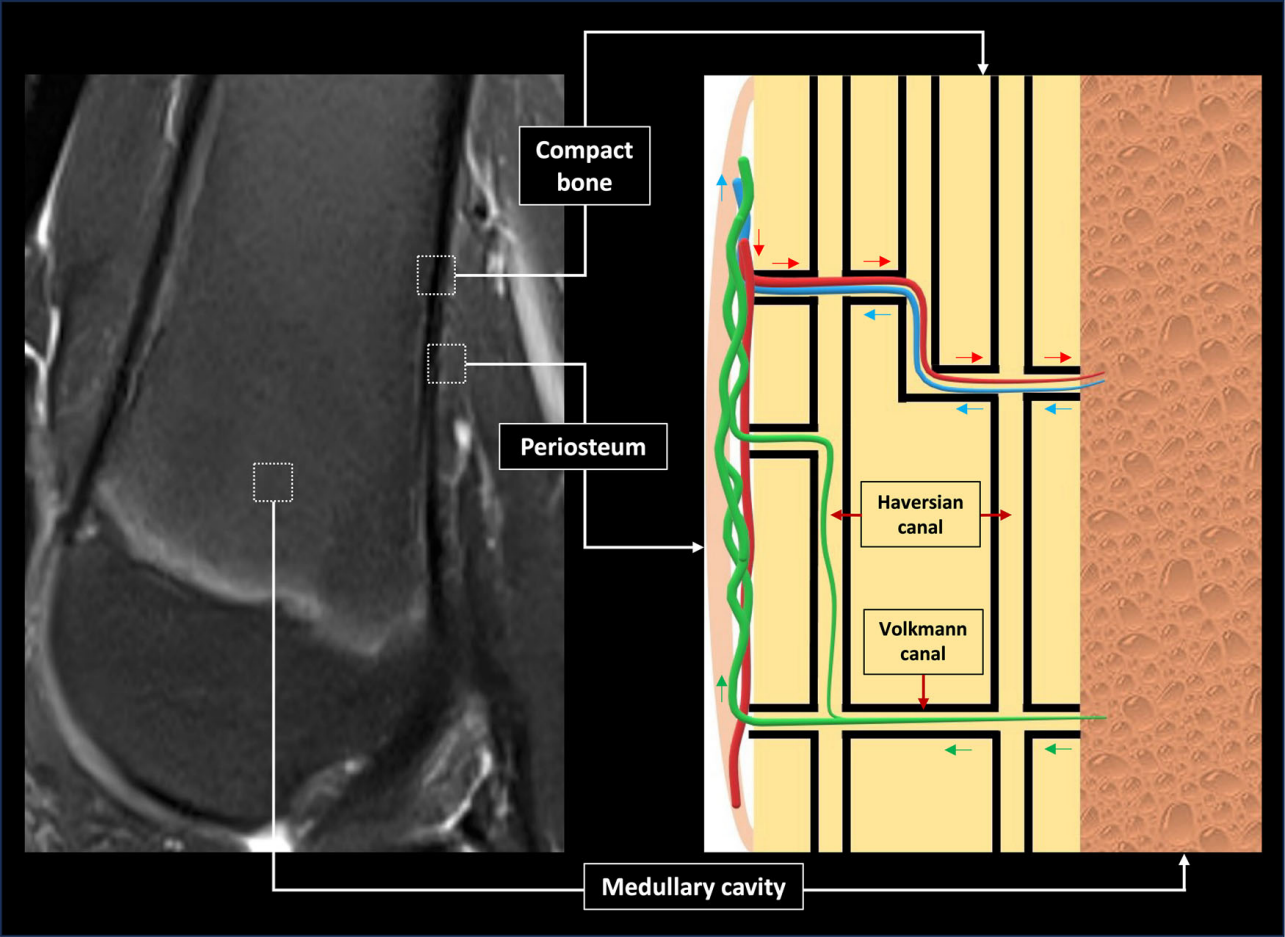
Bone lymphatic system anatomy. Different hypotheses exist regarding bone lymphatic system anatomy. In order to explain the images exhibited in this article, we hypothesize that intramedullary and intracortical lymphatics exist and progress along the bone arteries and veins through Haversian and Volkmann canals

The intraosseous lymphatic system is superimposed on the arterial and venous vascularization of the bone [7, 8]. Lymphatics follow arteries and veins, departing from the bone marrow and crossing the cortical bone through Volkmann and Haversian canals (Fig. 1) to join the periosteal lymphatics.

Normal intraosseous lymphatics are not seen on MRI and NEMRL. In lymphatic disorders with bone involvement, MRI and NEMRL can identify intraosseous dilated lymphatic vessels (lymphangiectasis). In this case, pathological lymphatics proliferate abnormally and dilate to become visible on imaging.

## Lymphatic diseases associated with skeletal involvement

Lymphatic skeletal lesions can be associated with diffuse lymphatic diseases, including GLA and primary lymphangiectasia [9]. These diseases are responsible for primary lymphatic system dysfunction and are caused by impaired lymphangiogenesis [9, 10]. In both diseases, there is an abnormal anarchic proliferation of lymphatic endothelial cells, resulting in numerous abnormal lymphatics developing along the lymphatic pathway. Numerous genetic mutations associated with these diseases have been identified [11].

In 2014, the International Society for the Study of Vascular Anomalies updated its classification for lymphatic vascular malformations, which can be found in Table 1 [12].

**Table 1:** Classification of lymphatic malformations (LM) according to the International Society for the Study of Vascular Anomalies (ISSVA)

Common (cystic) LM
Macrocystic LM
Microcystic LM
Mixed cystic LM
Generalized lymphatic anomaly
LM in Gorham–Stout disease
Channel-type LM
Primary lymphedema
Nonne-Milroy syndrome
Primary hereditary lymphedema
Lymphedema-distichiasis
Hypertrichosis-lymphedema-telangiectasia
Primary lymphedema with myelodysplasia
Microcephaly +/- chorioretinopathy, lymphedema, or mental retardation syndrome
Lymphedema-choanal atresia

GLA, formerly known as lymphangiomatosis, covers a spectrum of pathologic entities defined by the presence of cystic or noncystic lymphangiomas associated with anarchic proliferation of lymphatics called lymphangiectasis [13]. In GLA, bone involvement may be the primary feature, as in Gorham–Stout disease, or a secondary feature, as in lymphangiomatosis, where it is just one of many sites of lymphatic anomalies. Gorham–Stout disease (or vanishing bone disease) is typically depicted as a dramatic focal bone destruction with associated soft-tissue mass [14]. Some cases may present with multiple areas of bone destruction [2]. Associated soft tissue and visceral lymphatic anomalies are usually present and prominent [15]. In lymphangiomatosis, bone lymphangiomas or other osseous anomalies may be present, often without severe involvement (rare cortical destruction). Hence, lymphangiomatosis and Gorham–Stout disease may be identified as the same disease (GLA) with a spectrum of intensity of the skeletal involvement.

The imaging features of bone involvement in lymphatic diseases reflect underlying histopathological and molecular abnormalities. Understanding these mechanisms is critical for accurate diagnosis and management. The pathological examination of bone lesions in GLA and primary lymphangiectasia typically reveals an abnormal proliferation of lymphatic endothelial cells, with dilated lymphatic channels infiltrating the bone marrow and cortical bone [2, 9, 10]. In Gorham–Stout disease, osteoclastic activity is increased, leading to progressive bone resorption and replacement by fibrous and lymphatic tissue. Histologically, cystic lymphangiomas are lined by a single layer of endothelial cells, while noncystic lymphangiomas and lymphangiectasis show a dense network of dilated lymphatic vessels, sometimes associated with fibrosis and hemorrhagic foci. Genetic studies have identified mutations in the PI3K/AKT/mTOR pathway, which play a key role in lymphatic endothelial cell proliferation and survival [9, 10]. These mutations are also implicated in the aggressive bone destruction observed in Gorham–Stout disease, through increased osteoclastic activity and impaired osteoblastic function. The presence of intralesional fat, when observed, corresponds to adipose tissue trapped within dilated lymphatic spaces.

To simplify this taxonomy and the description of the presented cases, we consider that the presence of lymphangiomas on NEMRL can differentiate between GLA and primary lymphangiectasia: GLA presents multiple lymphangiomas with or without lymphangiectasis; primary lymphangiectasia presents with lymphangiectasis without lymphangiomas.

## Nonenhanced magnetic resonance lymphography (NEMRL)

Multiple imaging techniques are available for the initial work-up of diffuse lymphatic diseases. These different techniques, with their advantages and disadvantages, are described in Table 2.

**Table 2:** Different imaging techniques for the initial work-up diffuse lymphatic diseases

	NEMRL^a^	CT	Scintigraphy/SPECT-CT	Lymphography
Technique	No IV contrast	IV contrast (iodine)	Subcutaneous injection of Tc^99m^	Intranodal (inguinal) injection of ethiodized oil
PROS	Noninvasive Lymphatic mapping Lymphatic bone involvement visualization	Noninvasive Good spatial resolution Precise cortical and medullary bone analysis	Noninvasive Lymphatic mapping Lymphatic bone involvement visualization and cortical bone analysis, when coupled with CT	Good spatial resolution Lymphatic mapping Possible treatment
CONS	No dynamic study of lymphatic flow	Ionizing radiation Iodine injection Poor lymphatic visualization	Ionizing radiation Low spatial resolution (scintigraphy) Availability	Invasive Ionizing radiation Poor visualization of bone structures Availability

*NEMRL* nonenhanced magnetic resonance lymphography, *CT* computed tomography, *SPECT* single photon emission computed tomography

^a^ Magnetic resonance lymphography can also be performed after the injection of gadolinium in an inguinal lymph node (T1w sequences with dynamic acquisition)—this technique is of interest to visualize lymphatic leaks on dynamic post-contrast images

NEMRL consists of a free-breathing high spatial resolution fast-recovery fast spin-echo (FRFSE) sequence similar to 3D MR cholangiopancreatography [16–18]. 1.5-Tesla imaging is preferred over 3-Tesla in order to minimize motion artifact, susceptibility effect, and local field inhomogeneity, even if a higher signal-to-noise ratio is obtained at 3 Tesla. A large field of view is required, covering the chest and/or abdomen and pelvis, in coronal and sagittal planes. Additional acquisitions with fields of view centered on extremities may be necessary. Millimetric or submillimetric slice thickness is used. Scan time varies from 3 to 5 min, depending on the number of source images. Postprocessing of the data using maximum intensity projection (MIP), usually set at 20 mm thickness, is helpful for image analysis. Standard T2w fast spin-echo (FSE) is also acquired in the coronal and axial planes during the study. Technical parameters for NEMRL are presented in Table 3 [5].

**Table 3:** Technical parameters for NEMRL

Field strength	1.5 T
Sequence	3D high spatial resolution fast-recovery fast spin-echo (FRFSE)
Plane	Coronal/sagittal
TR (ms)	5000
TE (ms)	1000
Number of averages	1
Flip angle	90°
Matrix acquisition size	384 × 320
FOV (mm)	400 × 360
Number of slices	110–162
Slice thickness (mm)	1.4
Spacing (mm)	0
Gating	Free breathing with respiratory gating
Acquisition time (min)	5

While NEMRL is a valuable noninvasive tool for assessing lymphatic disorders, it has several limitations. The signal-to-noise ratio can be suboptimal, especially when millimetric or submillimetric slices are used. Furthermore, NEMRL does not provide functional information, such as lymphatic flow dynamics, which may require dynamic contrast-enhanced magnetic resonance lymphography that was recently introduced by injecting a gadolinium contrast agent into bilateral inguinal lymph nodes.

## NEMRL patterns in proliferative lymphatic diseases

On a normal NEMRL study, visceral lymphatics are identified as high–signal intensity thin linear structures easily recognized because of the characteristic alternating bands of constriction (lymphatic valves) with regular borders, and there are no lymphatics identified in bone structures [19]. In GLA and primary lymphangiectasia, NEMRL will identify numerous and dilated lymphatic vessels with ill-defined borders, called lymphangiectasis, located above and/or below the diaphragm. In GLA, lymphangiectasis can be associated with lymphangiomas. Cystic lymphangiomas (CL) are identified as high–signal intensity homogeneous cyst-like structures. Noncystic lymphangiomas (NCL) are identified as lymphangiectasis organized as a mass.

## Imaging patterns of skeletal involvement in diffuse lymphatic diseases

Intraosseous lymphatic anomalies usually present as nonspecific osteolytic bone lesions. They can affect axial or peripheral bones [2]. They do not always contain intralesional fat. In the presence of intralesional fat, bone hemangioma is the main differential. Intraosseous lymphatic anomalies may or may not be responsible for cortical bone destruction. A soft-tissue mass can sometimes be associated, especially in cases where bone destruction is present. When cortical destruction occurs, the main differentials include bone metastasis, multiple myeloma, and aggressive primary bone tumors. NEMRL is a useful additional diagnostic tool to identify extraosseous lymphatic anomalies, and sometimes a lymphatic “connection” between the intraosseous and the extraosseous lymphatic anomalies, in the setting of diffuse lymphatic diseases. Features of the different imaging patterns of bone involvement from our experience are summarized in Table 4.

**Table 4:** Features of different imaging patterns of skeletal involvement in diffuse lymphatic diseases

	Cystic lymphangioma	Noncystic lymphangioma	“Amorphous” lesions	Lymphangiectasis	Bone destruction
Number of lesions	Single or multiple				Usually single
Shape	Round/oval	Round/oval	No clear shape	Linear/snaking	Mass-like
Intralesional fat	+/-				
Lymphatic “connection” on NEMRL	Usually not visualized	Sometimes visualized	Usually not visualized	Sometimes visualized	Sometimes visualized
Cortical destruction	-	+/-	+/-	+/-	+++
Associated extraosseous lymphatic anomalies on NEMRL	+				

### Cystic lymphangioma (CL)

**Fig. 2:**
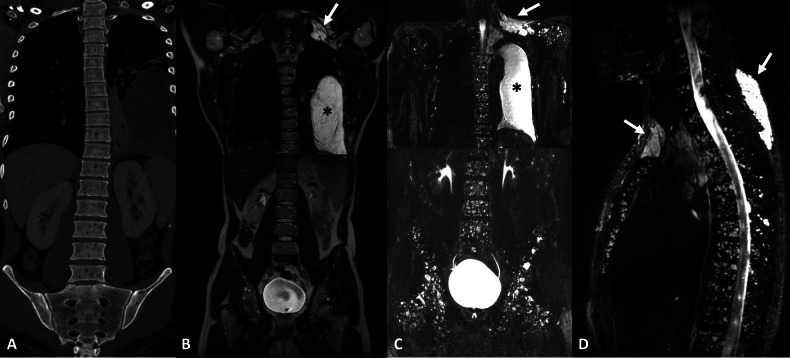
Initial work-up of a posterior cervical mass in a 26-year-old male revealing lymphangiomatosis (GLA) with multiple intraosseous cystic lymphangiomas of the axial skeleton. These multiple bone lesions did not cause any pain. **A** Post-contrast CT, coronal view. Multiple round lytic bone lesions, with regular and well-defined borders, without cortical destruction, located on the axial skeleton. **B** T2w TSE, coronal view. The multiple intramedullary lesions seen on CT exhibit homogeneous high signal intensity. There is no associated bone marrow edema. **C**, **D** 3D FRFSE T2w, MIP reconstruction (20 mm), coronal (**C**) and sagittal (**D**) views. NEMRL reveals associated lymphatic anomalies, including soft-tissue cystic lymphangiomas (arrows) of the left supra-clavicular space, superior mediastinum, posterior cervical area, and the left chylothorax (*). Note how the countless intraosseous cystic lymphangiomas are also well identified

**Fig. 3:**
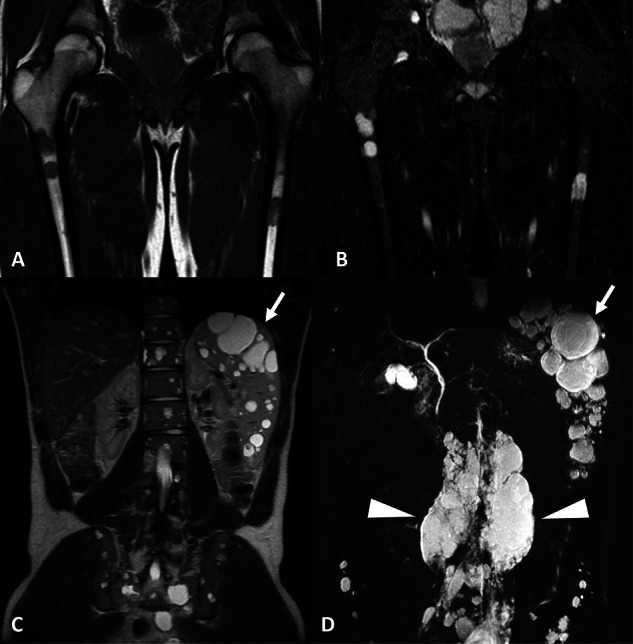
Follow-up of lymphangiomatosis (GLA) in an asymptomatic 18-year-old male with intraosseous cystic lymphangiomas of the peripheral skeleton. **A** T1w, coronal view. Bilateral diaphyseal intramedullary bone lesions, exhibiting low signal intensity with intralesional high signal intensity spots, demonstrating intralesional fat. **B** STIR, coronal view. These lesions exhibit high signal intensity. There is no cortical destruction and no soft-tissue mass. **C** T2w, TSE, coronal view. Multiple splenic nodular lesions with high signal intensity consistent with splenic lymphangiomas (arrow). Cystic lymphangiomas of the axial skeleton are also identified. **D** 3D FRFSE T2w, MIP reconstruction (20 mm), coronal view. NEMRL reveals associated lymphatic anomalies, including bulky retroperitoneal cystic lymphangiomas (arrowheads) and splenic lymphangiomas (arrow)

Cystic lymphangioma (CL) is a focal dilation of a lymphatic vessel, filled with lymphatic fluid. CLs are described as cyst-like lesions on imaging because they resemble classic cysts. They may be encountered anywhere along the lymphatic pathways, and their communication with lymphatic vessels is seldom identified. They can be unique or multiple. CLs are round or oval-shaped, with regular and well-defined borders. They usually present with a low-intensity signal on T1-weighted (T1w) images and a high-intensity signal on T2-weighted (T2w) images. On CT, CLs present as a low-attenuating lytic bone lesion. CLs may contain fat, but this feature is inconstant [20]. When they do, they may exhibit areas of intermediate-intensity or high-intensity signal on T1w images. When present, they usually involve the axial skeleton and, more rarely, the peripheral skeleton. They are intramedullary lesions and are not associated with cortical destruction. In GLA, lymphangiomas are usually multiple and associated with noncystic lymphangiomas and lymphangiectasis (Figs. 2, 3), which are easily revealed with NEMRL. Lymphangiomas are typically not present in primary lymphangiectasia.

### Noncystic lymphangioma (NCL)

**Fig. 4:**
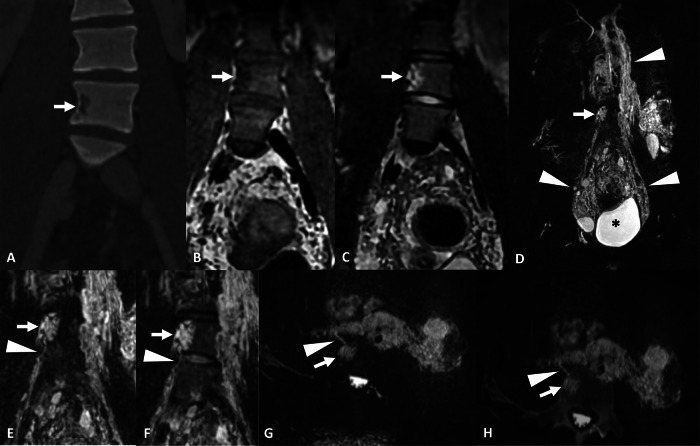
Initial work-up of a buttock and thigh swelling in a 16-year-old male revealing GLA with intraosseous noncystic lymphangioma. **A** Noncontrast CT, coronal view. Juxtacortical lesion of vertebra with low attenuation density and without cortical destruction (arrow). **B**, **C** T1w and STIR, respectively, coronal view. Juxtacortical lobulated vertebral lesion, with irregular borders, low signal intensity on T1w images and high signal intensity on STIR images. Note the few high signal intensity spots on T1w images disappearing on STIR images corresponding to intralesional fat (arrow). **D** 3D FRFSE T2w, MIP reconstruction (20 mm), coronal view, revealing a bulky pelvic lymphangioma (*) associated with retroperitoneal and pelvic lymphangiectasis (arrowheads). Note the vertebral noncystic lymphangioma (arrow). **E**, **F** 3D FRFSE T2w, MIP reconstruction (20 mm), coronal view and fusion between NEMRL and STIR image. Identifies a connection (arrowhead) between the vertebral noncystic lymphangioma (arrow) and the paravertebral retroperitoneal lymphangiectasis. In the presence of a primary lymphatic disease, characterized by lymphatic proliferation, the connections between the intraosseous and extraosseous lymphatics become hyperplastic and dysplastic and hence visible on NEMRL. **G**, **H** 3D FRFSE T2w, MIP reconstruction (20 mm), axial view and fusion between NEMRL and noncontrast CT image. The connection (arrowhead) between the vertebral noncystic lymphangioma (arrow) and the paravertebral retroperitoneal lymphangiectasis is also well visualized on axial images. Note the paravertebral retroperitoneal lymphangiectasis sheathing the inferior vena cava and the abdominal aorta

Noncystic lymphangioma (NCL) is a pool of lymphangiectasis organized as a mass. They are usually lobulated with ill-defined borders. Small linear intralesional high-intensity structures can be identified on T2w images, corresponding to small lymphangiectasis. They may or may not contain fat. On CT, NCLs present as a low-attenuating lytic bone lesion. They may be intramedullary or juxtacortical. Cortical destruction is usually not associated. The communication with lymphatic vessels can be clearly identified with NEMRL (Fig. 4) but is inconstant. Like CLs, they are usually associated with lymphangiectasis in GLA but are not seen in primary lymphangiectasia. NEMRL reveals associated extraosseous lymphatic anomalies such as lymphangiomas and lymphangiectasis.

### “Amorphous” or regional lesions

**Fig. 5:**
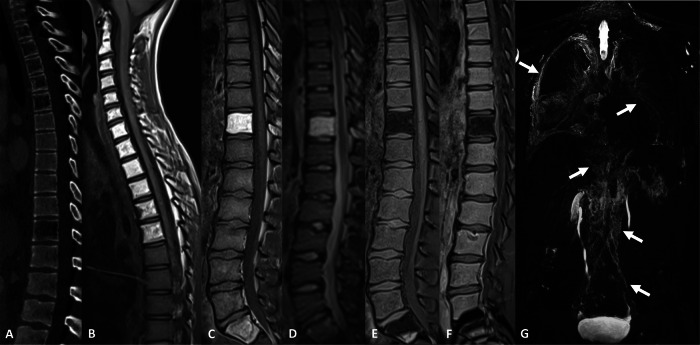
Bone marrow heterogeneity discovered on chest CT performed for recurring pulmonary infections in an 11-year-old male, revealing primary diffuse lymphangiectasis. **A** Noncontrast CT, sagittal view. Areas of osteolysis with fat attenuation density involving multiple vertebral bodies, without cortical destruction. **B**, **C** T1w, sagittal view. Corresponding anomalies demonstrating high signal intensity on a T1w image, corresponding to intralesional fat. **D** T2w, sagittal view. Anomalies also exhibit high signal intensity on T2w images. **E**, **F** T1w DIXON, water, before and after contrast administration, respectively, sagittal view. Absence of intralesional enhancement. Differentials include vertebral body hemangioma. **G** 3D FRFSE T2w, MIP reconstruction (20 mm), coronal view. Reveals supra- and infradiaphragmatic lymphangiectasis (arrows), including subpleural, intrapulmonary, retroperitoneal, and pelvic lymphangiectasis. The associated diffuse lymphangiectasis orients the vertebral anomalies to be of lymphatic origin

**Fig. 6:**
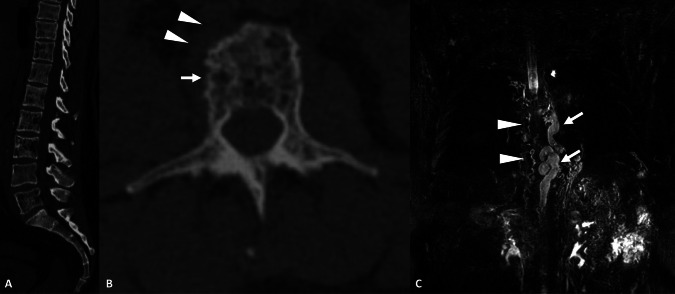
Work-up of osteolytic lesions of the spine in a 62-year-old female revealing primary diffuse lymphangiectasis. **A** Noncontrast CT, sagittal view. Diffuse patchy osteolytic lesions of the spine demonstrating fat attenuation density. **B** Noncontrast CT, axial view. Areas of cortical erosions with a moth-eaten appearance (arrow). A nonspecific prevertebral tissular mass is also identified (arrowheads). **C** 3D FRFSE T2w, MIP reconstruction (20 mm), coronal view revealing paravertebral lymphangiectasis (arrowheads), corresponding to the prevertebral tissular mass on CT, and a massive dilation of the thoracic duct (arrows)

“Amorphous” or regional anomalies encompass the entirety or an area of a bone piece. They can be found in both GLA and primary lymphangiectasia. Vertebral bodies are often involved. These anomalies present as areas of osteolysis. They may or may not contain fat. When intralesional fat is present, it can be challenging to differentiate it from hemangiomas (Fig. 5) [21]. On standard MRI, they present a variable signal in T1w images according to the presence on intralesional fat. They present a high signal intensity on T2w images. They may or may not be enhanced after contrast administration. They can be associated with cortical osteolysis (Fig. 6). Periosseous fat infiltration or a soft-tissue mass can sometimes be identified on standard CT and MRI, corresponding to dilated lymphatics. On NEMRL, they are identified as intraosseous areas of high signal intensity as the sequences are T2w. NEMRL also exhibits associated extraosseous lymphatic anomalies such as lymphangiomas and lymphangiectasis.

### Lymphangiectasis

**Fig. 7:**
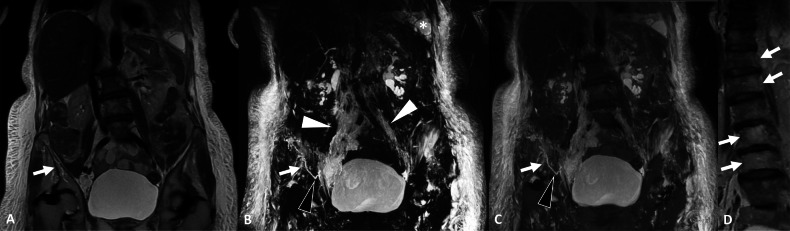
Initial work-up of a chylothorax of unknown origin in a 75-year-old male presenting with shortness of breath, revealing primary diffuse lymphangiectasis. **A** T2w, TSE, coronal view. Linear snaking lesion of the right iliac bone (arrow), without cortical destruction or soft-tissue mass. **B** 3D FRFSE T2w, MIP reconstruction (20 mm), coronal view. NEMRL reveals diffuse lymphatic anomalies with retroperitoneal and pelvic lymphangiectasis (white arrowheads). There is no identified lymphangioma. Massive bilateral subcutaneous lymphedema and left-sided chylothorax (*) are associated. NEMRL brings to light a connection between the iliac intraosseous lymphangiectasis and the pelvic lymphangiectasis (black arrowhead). **C** Fusion between NEMRL and T2w images. Fusion of the two images enables superposition of the lymphatic pathways on an anatomical image and helps identify the connection (black arrowhead). **D** T2w, coronal view. Coexistence of “amorphous” lesions of several vertebral bodies, ill-defined and with high signal intensity on T2w images, corresponding to intraosseous lymphatic anomalies

**Fig. 8:**
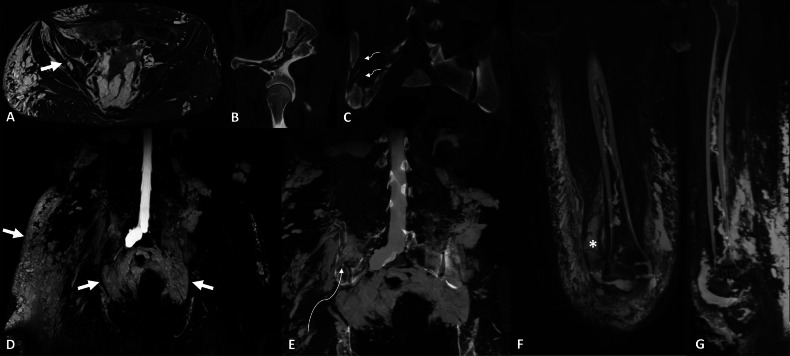
Work-up for recurrent septic arthritis of the right knee and right buttock swelling in a 35-year-old male. **A** STIR, axial view. Linear snaking lesion (arrow) of the right iliac bone with high signal intensity on T2w images. **B**, **C** Noncontrast CT, sagittal oblique (**B**) and coronal (**C**) views. Corresponding anomalies with low attenuation density. The “point of entry” of the lymphangiectasis (curved arrows) in the iliac bone is well identified on the coronal view. **D** 3D FRFSE T2w, MIP reconstruction (20 mm), coronal view. NEMRL reveals diffuse lymphatic anomalies with pelvic and subcutaneous lymphangiectasis (arrows). These findings are compatible with diffuse primary lymphangiectasis. **E** Fusion of NEMRL and CT images, coronal view. NEMRL brings to light a connection between the pelvic lymphangiectasis and intraosseous lymphangiectasis (curved arrow) through the point of entry identified on CT. **F**, **G** NEMRL of the lower extremities reveals intraosseous lymphangiectasis of the femoral diaphysis and of surrounding soft tissues. Fluid effusion is also identified (*). Impaired lymphatic drainage of the right lower extremity is most likely responsible for the recurring septic arthritis of the knee

Lymphangiectasis are dilated lymphatic vessels with ill-defined borders. They can be found in both GLA and primary lymphangiectasia. They present as linear lytic bone lesions with low attenuation and sclerotic margins on CT. On MRI, they are identified as linear structures with low signal intensity on T1w images and high signal intensity on T2w images. Lymphangiectasis in bones is clearly identified with NEMRL. Furthermore, NEMRL can reveal the connection between a lymphangiectasis and the extraosseous lymphatics. Because the lymphangiectasis enters the bone in one spot, “points of entry” with cortical lysis are usually identified using CT (Figs. 7, 8). They can be seen in both GLA and primary lymphangiectasia.

### Bone destruction

Bone destruction corresponds to osteolytic lesions with cortical loss. This pattern is associated with the typical Gorham–Stout disease. Although cortical destruction is the hallmark of Gorham–Stout disease, it can also be identified in GLA and other primary lymphangiectasia. Gorham–Stout disease belongs to the spectrum of GLA with bone involvement, where the bone involvement is in the foreground and preponderant.

**Fig. 9:**
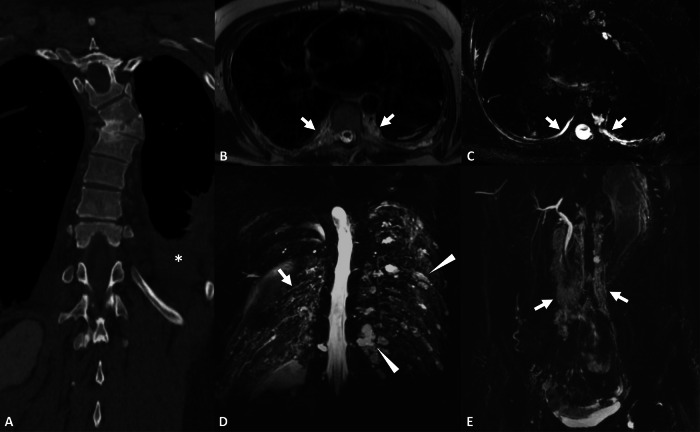
Vertebral compression fracture in a 38-year-old female patient with a history of unexplained chylothorax, revealing GLA with bone destruction (Gorham–Stout disease). **A** Noncontrast CT, coronal view. Osteolysis of the right half of the Th7 vertebral body, with pseudo-fracture aspect, responsible for deformity in the coronal plane with lateral inflexion. **B** T2 TSE, axial view. Paravertebral and intercostal infiltration demonstrating high-intensity signal on T2w images (arrows). **C** 3D FRFSE T2w, MIP reconstruction (20 mm), axial view. Reveals paravertebral lymphangiectasis (arrows) corresponding to paravertebral infiltration on T2w images. **D**, **E** 3D FRFSE T2w, MIP reconstruction (20 mm), coronal view. NEMRL reveals additional intercostal lymphangiectasis (arrow) and intercostal lymphangiomas (arrowheads). More lymphatic anomalies are identified below the diaphragm, with retroperitoneal lymphangiectasis (arrows)

**Fig. 10:**
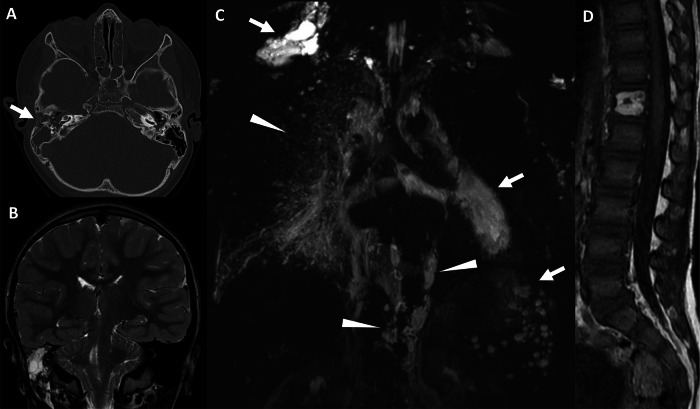
Recurrent meningitis in a 4-year-old boy revealing GLA with bone destruction (Gorham–Stout disease). **A** Noncontrast CT, axial view. Osteolysis of the right petrous apex with osteomeningeal fistula (arrow). **B** T2w, coronal view. Lobulated lesion of the right petrous apex with high signal intensity. **C** 3D FRFSE T2w, MIP reconstruction (20 mm), coronal view. NEMRL reveals diffuse lymphatic anomalies: supra-clavicular, mediastinal, and splenic lymphangiomas (arrows) associated with peribronchovascular and paravertebral lymphangiectasis (arrowheads). **D** T1w, sagittal view. Fat-containing amorphous lesion of the Th12 vertebral body with high signal intensity

Classical Gorham–Stout disease involves a focal progressive bone destruction and resorption, replaced by a soft-tissue mass corresponding to angiomatosis of blood and lymphatic vessels. This results in an image of a disappearing bone on standard radiography. Osteolytic lesions with cortical interruption are well identified on CT. MRI can identify the soft-tissue mass developing inside and around the bone, which usually has high signal intensity on T2w images [15]. Associated lymphatic anomalies such as lymphangiomas, lymphangiectasis, and amorphous regional lymphatic anomalies can be identified with NEMRL (Figs. 9, 10). There is a large polymorphism in the expression of lymphatic bone lesions that can range from purely asymptomatic lesions to lesions with very pronounced clinical expression. Gorham–Stout syndrome is characterized by progressive, destructive osteolysis of a bone segment, often accompanied by significant clinical symptoms such as pain, deformity, or pathological fractures. In contrast, asymptomatic lymphatic bone lesions lack this aggressive bone destruction and typically present as incidental radiographic findings without functional impairment.

## Value of NEMRL in undetermined osteolytic bone lesions

In our experience, NEMRL is a useful tool with which to investigate the lymphatic origin of a bone lesion. In cases of destructive osteolytic bone lesions, differential diagnoses include metastatic disease, multiple myeloma, and primary bone tumors. Diagnosis can be challenging, and NEMRL can provide useful information to suggest a lymphatic cause. Indeed, associated diffuse visceral lymphatic anomalies are easily identified with NEMRL. Identifying these visceral anomalies when facing an undetermined bone lesion may guide diagnosis toward a lymphatic cause. Furthermore, NEMRL sometimes identifies a lymphatic connection between the bone lesion and the extraosseous lymphatic system or anomalies, confirming lymphatic impairment to be the cause of the bone lesion.

## Complications of bone involvement in diffuse lymphatic diseases

Intraosseous lymphatic proliferation may modify and weaken bone structure, increasing the risk of pathological bone fracture [22]. Bone pain and deformities have also been described [2]. Local structure compression and invasion by extensive bone lesions may lead to specific symptoms such as radiculopathy [23].

The lymphatic system plays a role in the defense against infections. Lymphocytes and immunoglobulins present in lymphatic fluid actively participate in specific immune responses and are bacteriostatic [24]. Impaired intraosseous lymphatics may favor the development of septic arthritis and osteomyelitis [25, 26].

## Treatment

There is no consensus treatment for diffuse lymphatic diseases, and treatment most often focuses on complications. Multiple therapeutic options are available, including medical therapy, interventional radiology, radiation therapy, and surgery [27].

Medical therapy of diffuse lymphatic diseases usually begins with conservative management aiming at decreasing lymphatic fluid production by reducing dietary fat intake.

Medical therapy with m-TOR inhibitors (sirolimus) inhibits lymphangiogenesis and may be offered in some cases [9].

Specific treatments for lymphatic osteolytic bone lesions include drug therapy with bisphosphonates, which inhibit osteolysis. Pathological fractures, including vertebral fractures, may be accessible to percutaneous cementoplasty and vertebroplasty. In large osteolytic lesions, the use of embolization or radiotherapy has been reported. Surgery can be considered when nearby organs are being compressed [28–30].

All treatments are discussed on a case-by-case basis. Ozeki and al. have proposed a therapeutic algorithm for cases presenting with bone involvement [9]. Pathological fractures and deformations, including scoliosis, warrant initial orthopedic surgery. Patients with progressive osteolytic lesions should be offered bisphosphonate therapy and eventually radiotherapy if progression is ongoing. Asymptomatic patients with bone involvement on imaging should be observed and eventually treated if progression occurs or symptoms appear.

## Conclusion

NEMRL is a useful noninvasive and non-ionizing tool to assess skeletal and visceral involvement in diffuse lymphatic diseases. Imaging features may help in diagnosis and differentials and guide patient management.

## Data Availability

All data displayed in the article, including images, are available on our local picture archiving and communication system.

## Abbreviations

CL: Cystic lymphangioma
CT: Computed tomography
GLA: Generalized lymphatic anomalies
MRI: Magnetic resonance imaging
NCL: Noncystic lymphangioma
NEMRL: Nonenhanced magnetic resonance lymphography
